# Beyond glucose: alternative sources of energy in glioblastoma

**DOI:** 10.7150/thno.53506

**Published:** 2021-01-01

**Authors:** John L. Caniglia, Anvesh Jalasutram, Swapna Asuthkar, Joseph Sahagun, Simon Park, Aditya Ravindra, Andrew J. Tsung, Maheedhara R. Guda, Kiran K. Velpula

**Affiliations:** 1 Department of Cancer Biology and Pharmacology, University of Illinois College of Medicine, Peoria.; 2 Department of Neurosurgery, University of Illinois College of Medicine at Peoria.; 3 Department of Pediatrics, University of Illinois College of Medicine at Peoria.; 4 Illinois Neurological Institute, Peoria, IL.

**Keywords:** glioblastoma, fatty acids, metabolism, arginine, glutamine, autophagy

## Abstract

Glioblastoma multiforme (GBM) is the most common malignant brain tumor in adults. With a designation of WHO Grade IV, it is also the most lethal primary brain tumor with a median survival of just 15 months. This is often despite aggressive treatment that includes surgical resection, radiation therapy, and chemotherapy. Based on the poor outcomes and prevalence of the tumor, the demand for innovative therapies continues to represent a pressing issue for clinicians and researchers. In terms of therapies targeting metabolism, the prevalence of the Warburg effect has led to a focus on targeting glucose metabolism to halt tumor progression. While glucose is the dominant source of growth substrate in GBM, a number of unique metabolic pathways are exploited in GBM to meet the increased demand for replication and progression. In this review we aim to explore how metabolites from fatty acid oxidation, the urea cycle, the glutamate-glutamine cycle, and one-carbon metabolism are shunted toward energy producing pathways to meet the high energy demand in GBM. We will also explore how the process of autophagy provides a reservoir of nutrients to support viable tumor cells. By so doing, we aim to establish a foundation of implicated metabolic mechanisms supporting growth and tumorigenesis of GBM within the literature. With the sparse number of therapeutic interventions specifically targeting metabolic pathways in GBM, we hope that this review expands further insight into the development of novel treatment modalities.

## Introduction

Glioblastoma multiforme (GBM) is the most common primary brain malignancy in adults, causing a yearly average of 3.19 new cases per 100,000. It is also the most lethal primary brain malignancy, causing a 2-year survival rate of 26-33%, a 4-5% survival rate at 5 years 1, 2, and a median survival time of just 15 months. It should be noted that the abysmal survival with GBM is often in spite of aggressive, multimodal treatments involving chemotherapy, radiation therapy, and immunotherapy in tandem with surgical resection 2. Thus, as can be seen from the prevalence and poor outcomes associated with GBM, there is a pressing demand for new and innovative therapies for both tumor prevention and treatment, and one such avenue of research involves targeting GBM metabolism.

In terms of therapies targeting metabolism, the prevalence of the Warburg effect characterized by cancer cell glycolysis instead of mitochondrial oxidative phosphorylation 3, has led to a focus on targeting glucose metabolism to halt tumor progression. While glucose is the dominant energy source in GBM, a number of metabolic pathways specifically exploited in cancer, are also prevalent in GBM in order to meet the demand by rapidly proliferating tumor. Namely, these pathways include fatty acid oxidation, the urea cycle, the glutamate-glutamine cycle, and cellular autophagy.

The goal of this review is to develop core knowledge regarding the metabolic processes implicated in tumor differentiation and proliferation - the basis from which further efforts to target and impede GBM metabolism can proceed. Given that the targeting of tumor metabolism is not a part of the current battery of treatments for GBM utilized in clinical practice today, this vein of research will hopefully engender bench-to-bedside progress and uncover novel, practical, and effective treatment options for those suffering from GBM and improve both quality of life and survival times for patients.

## Glycolysis

A discussion of alternative methods of GBM metabolism cannot be had without first discussing the preferred method of GBM metabolism: glycolysis. One of the earliest described abnormalities of a metabolic pathway in cancer was within glycolysis, namely the Warburg effect. The Warburg effect refers to the discovery by Otto Warburg that cancerous cells utilized high amounts of glucose while excreting substantial amounts of lactate even in the presence of oxygen 4. He later hypothesized that mitochondrial dysfunction within malignant cells led to an abnormal dependence on anaerobic metabolism to meet energy demands. A variety of cancers, including GBM, does in fact utilize this process to meet their metabolic demands 5. Although the ubiquity of the Warburg effect has come into question recently, it has been shown more specifically that the tumor microenvironment determines whether cells will rely primarily on glycolysis or oxidative phosphorylation 6, 7. Under hypoxic microenvironments such as within its necrotic core, a majority of surrounding cells will utilize only glycolysis, while those near the vasculature perform oxidative phosphorylation due to increased oxygen concentration 7. It is worth noting that it is unknown if hypoxia regulates other metabolic aspects in GBM. However, this preference for glycolysis in GBM likely derives from its utility as a rapid source of ATP compared to other pathways 6. Nonetheless, further studies are warranted to determine if any preference for a specific metabolic mechanism exists within GBM.

With a high reliance on glycolysis in some tumor microenvironments, a number of therapeutics aimed at reversing the Warburg effect are being developed in efforts to prevent GBM growth and proliferation 8. In a study by Poteet et al., researchers demonstrated the use of methylene blue in reversing the Warburg effect in GBM through accepting electrons from NADH in mitochondrial complex I and transferring them to cytochrome c, thus shunting pyruvate into the citric acid cycle. When combined with temozolomide, the effects of methylene blue were additive in both sensitized and resistant temozolomide GBM cell lines U87 and T98G, respectively 9. In another study, Velpula et al. report that targeting of pyruvate dehydrogenase kinase (PDK1) reverses the Warburg effect by decreasing hypoxia-inducible factor (HIF-1⍺) expression within GBM 10. The hypoxic core of GBM stabilizes HIF-1⍺ expression, which induces activation of PDK1, and epidermal growth factor receptor (EGFR). By targeting PDK1 the metabolic preferences of GBM cells switch from predominantly glycolysis toward oxidative phosphorylation, leading to apoptosis, anti-proliferation effects, and reducing invasive capabilities within the U251 and 5310 cell lines 10. Other proteins implicated in the propagation of the Warburg effect include upregulation of glucose transporter 1 (GLUT1), hexokinase 2 (HK2), M2 isoform of pyruvate kinase (PKM2), and lactic acid dehydrogenase A (LDHA) 8. The literature demonstrates that targeting any of these proteins reverses the Warburg effect in GBM, inducing apoptosis and reactive oxygen susceptibility.

In summary, the glycolytic mechanisms are the most primitive pathways for energy but the most favorable for sophisticated cancers such as GBM. The regulation of glycolysis is an intricate interplay involving GLUT1, HK2, PKM2, LDHA, HIF-1⍺, tumor microenvironment, and mitochondrial genes 8-10. The current literature has demonstrated that glycolysis is a significant therapeutic target for GBM. Further studies are necessary to clarify and optimize anti-glycolytic therapy with adjunctive temozolomide, including GBM subtype dependence on glycolysis and preferences of alternative metabolic mechanisms 11, 12. In summary, the implication of glycolysis in GBM energy metabolism extends beyond just an initial pathway for ATP. It serves as a central hub for various reactions that its intermediates may shunt out to supply other mechanisms, and other pathways may feed into it.

## Fatty Acid Oxidation

**Figure 1:**
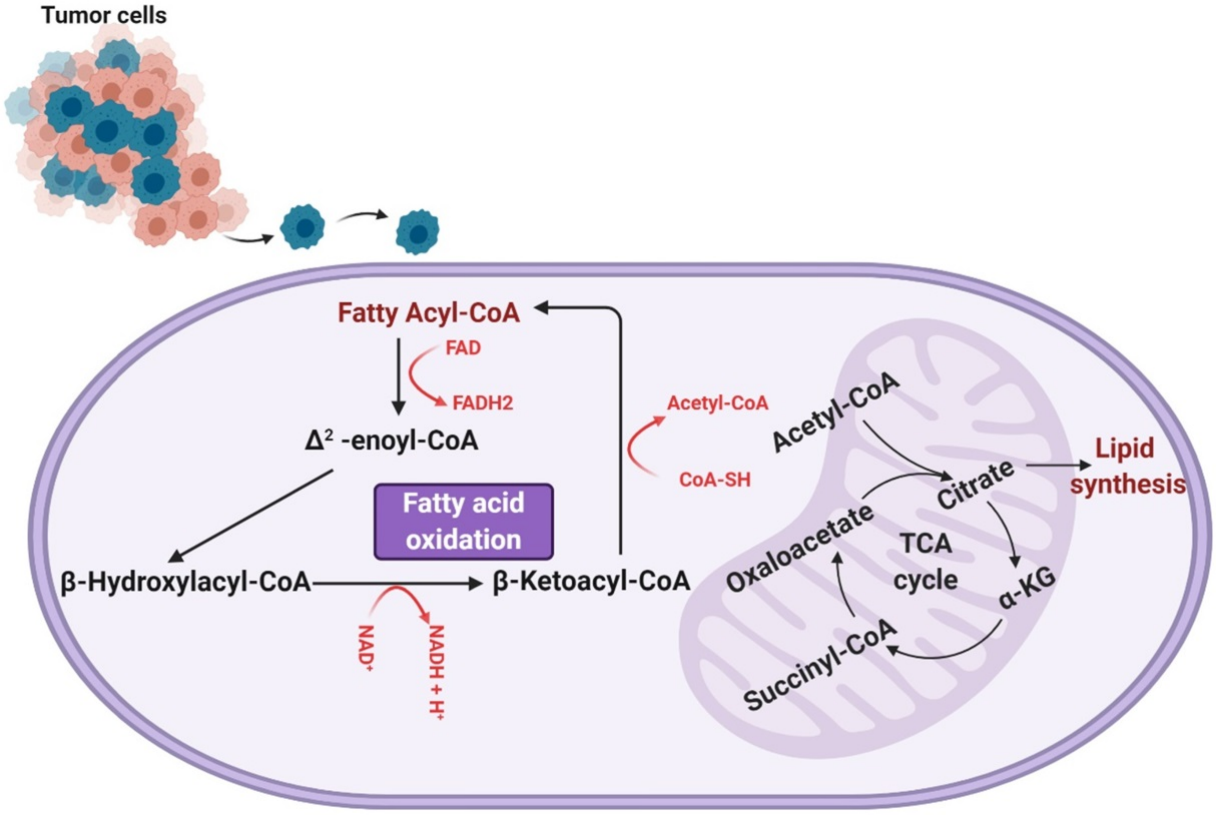
A representation of the process of fatty acid (beta) oxidation, which takes place within the mitochondrial matrix. Fatty acyl-CoA is initially converted to Δ^2^-enoyl-CoA, generating one molecule of FADH_2_. The Δ^2^-enoyl-CoA is converted to β-hydroxylacyl-CoA and then β-ketoacyl-CoA, producing an NADH. β-ketoacyl-CoA is then further processed to regenerate a fatty acyl-CoA (now two carbons shorter than when the process of fatty acid oxidation began) and produce an acetyl-CoA. This cyclic shortening repeats until the fatty acid has been completely consumed or, if the fatty acid was composed of an odd number of carbons, until the three-carbon structure propionyl-CoA is all that remains. It is worth noting that the process of fatty acid oxidation does produce any ATP itself, but rather produces metabolites that feed into other metabolic processes. Specifically, the FADH_2_ and NADH produced are shunted to the electron transport chain, the generated acetyl-CoA feeds into the tricarboxylic (TCA) cycle, and any propionyl-CoA produced is converted to succinyl-CoA to enter the TCA cycle as well.

Fatty acid oxidation involves a cyclic shortening of acyl-CoA molecules through the removal of two-carbon acetyl-CoA subunits and the generation of one NADH and one FADH 13. This shortening of the acyl-CoA is repeated either until the acyl-CoA is fully broken down or until it reaches a three-carbon structure known as propionyl-CoA, which can be converted to succinyl-CoA and used to further the TCA cycle (a separate mode of energy production). The units of NADH and FADH_2_ contribute to the electron transport chain in the mitochondria, which are consumed via oxidative phosphorylation for further ATP production 13. An overview of fatty acid oxidation can be seen in **Figure 1.**

As shown, fatty acid oxidation presents as an extremely useful source of energy, yielding more ATP than glycogenolysis by a factor of six per unit mass 13, 14. Further, the activity of fatty acid oxidation in GBM has been experimentally shown to contribute to aerobic respiration. Enzymes involved in fatty acid oxidation, particularly carnitine palmitoyltransferase and long-chain acyl-CoA dehydrogenase, have been found to be upregulated in human glioma tissue 15, 16. Lin et al. also determined the degree to which aerobic respiration in the mitochondria is dependent on fatty acids. Utilizing a Seahorse Analyzer to measure oxygen consumption rate (a measure of the oxygen-dependent metabolic activity occurring in a cell), a comparison of the oxygen consumption of hGBM treated with linoleic acid and etomoxir (to inhibit fatty acid oxidation) and that of hGBM treated with FCCP and antimycin A (to induce maximal oxidative respiration) was conducted, and the comparison revealed that a significant majority, nearly 80%, of oxygen respiration is dependent of fatty acid oxidation 15. This dependence of glioma on fatty acid oxidation was corroborated by Juraszek et al. and Fink et al., who both measured the expression of SLC22A5/OCTN2 (an organic transporter that delivers carnitine to a cell and is an important component of fatty acid oxidation) and found it to be overexpressed in human glioma cells 16, 17. In addition, they observed a decrease in viability and an increase in apoptosis in human glioma cells in response to inhibition of fatty acid oxidation 16, a finding that was also reported by Bi and colleagues 18. Thus, fatty acid oxidation contributes heavily to the generation of energy of gliomas and, as such, presents an avenue of research critical to our understanding of how gliomas subvert normal energy production in order to maintain aberrant growth and proliferation 14.

Experimental results have also precipitated the idea that fatty acid oxidation, in addition to functioning as a direct source of energy, also contributes to cancer survival by limiting the levels of reactive oxygen species (ROS) within cells, as uncontrolled increases in ROS levels can result in cancer cell death 19. Pike et al. analyzed the levels of intracellular ROS present in cells 25 minutes after treatment with 1 mM of the CPT-1 inhibitor etomoxir 19. Ultimately, it was found that, as hypothesized, that elevated levels of ROS (reflected in the elevated level of superoxide fluorescence) were found in cells treated with etomoxir when compared to control cells (i.e. cells not treated with etomoxir) 19.

## Urea Cycle

The urea cycle is often viewed as an intrinsic mechanism of metabolizing nitrogenous waste. With ammonia normally accumulating as a toxic byproduct of homeostasis, the urea cycle serves an integral role in its conversion to urea. However, it is of great importance to recognize that there are many intermediary reactions that comprise this biochemical pathway. Substrates heavily utilized in cancer metabolism are no exception 20. It is with this understanding that the urea cycle is discussed here from the perspective of tumor metabolism.

Arginine is well described as a substrate of many metabolic functions that become upregulated in cancer cells. Its roles in anabolic activities in nitric oxide, protein, and polyamine synthesis have been well described within the available literature. It is through many of these pathways that arginine has been described to promote cancer activity in angiogenesis and tumor growth 21. It is with that understanding that arginine is often described as conditionally essential due to the increased demand observed in many cancer phenotypes 22. Readily available under physiologic conditions, many solid tumors such as GBM demonstrate an elevated utilization of arginine that manifests as an extrinsic dependence 21. As traditional sources of arginine such as the urea cycle become overwhelmed, maintenance of adequate sourcing heavily relies upon uptake from the extracellular environment. Interestingly, certain phenotypes of GBM have been described to demonstrate a reduced capacity for endogenous arginine synthesis 23. Through epigenetic silencing of argininosuccinate synthetase 1 (ASS1), the urea cycle has been described to become effectively inhibited as the ASS1 gene product is responsible for catalyzing the rate limiting step 23. Consequently, these cancer phenotypes display an auxotrophic behavior that further facilitates dependence on extracellular arginine 24.

Although the underlying functions are not entirely understood, ASS1 silencing has been described to confer many biochemical advantages in the cancer phenotypes that display this behavior 23. Inhibition of ASS1 has been well described as an effective mechanism to shunt upstream substrates to other metabolic pathways. Rabinovich et al previously hypothesized that ASS1 silencing under normoxic conditions promoted anabolic functions essential to cellular proliferation 25. With aspartate serving as a substrate of both arginine and pyrimidine synthesis, the downregulation of ASS1 was hypothesized to shunt the amino acid towards *de novo* pyrimidine synthesis. This was further supported by demonstration of increased CAD (carbamoyl-phosphate synthetase 2, aspartate transcarbamylase, and dihydroorotase) activity in ASS1 silencing cancer cells 25. Serving as a trifunctional enzyme, CAD largely regulates the initiation of de novo pyrimidine synthesis. Its upregulation in activity was thought to result from increasing the cytosolic concentration of aspartate, in addition to mammalian target of rapamycin (mTOR) signaling. The study further elucidated this association by demonstrating the suppressive effects that CAD inhibition holds on the proliferation of ASS1 silencing cells 25.

**Figure 2:**
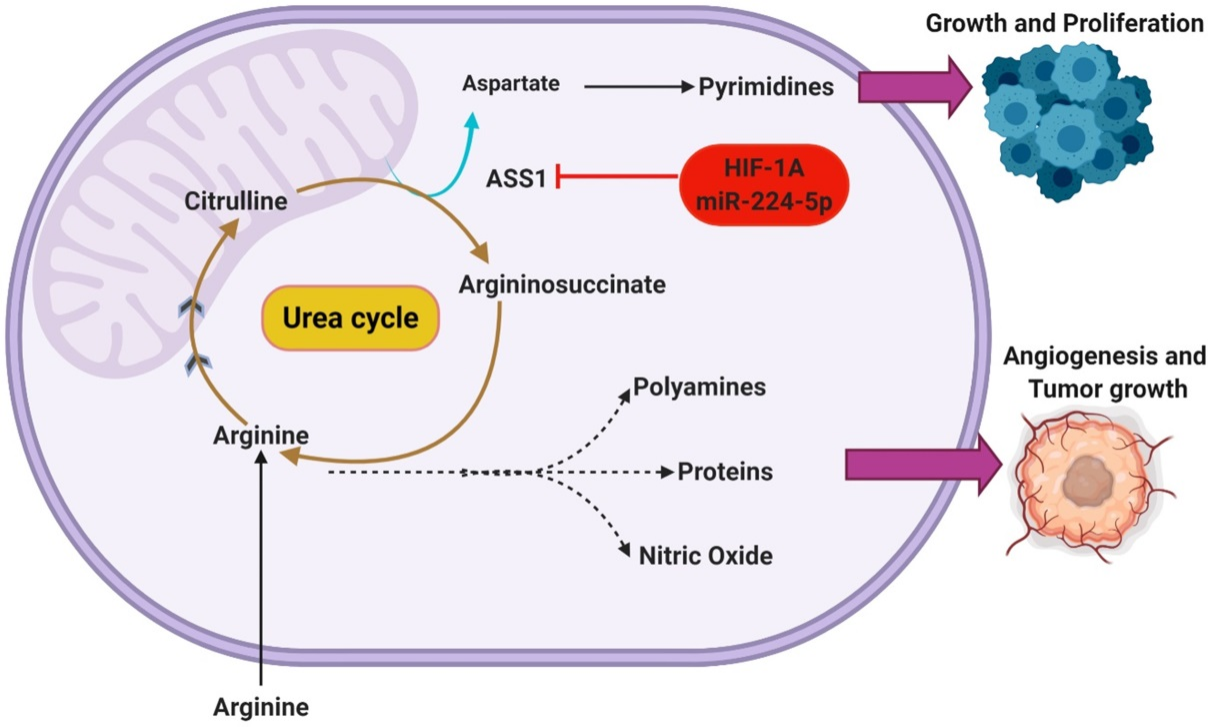
Silencing of ASS1 occurs in malignant GBMs through HIF-1α activation or epigenetic mechanisms. This functionally inhibits the urea cycle, shunting key metabolic intermediates toward other pathways. The amino acid arginine can be used in the biosynthesis of nitric oxide to support angiogenesis or can be incorporated in polyamines and proteins to support tumor growth. Additionally, the accumulation of aspartate in ASS1-silenced tumors supports the continued synthesis of pyrimidine nucleotides.

It is important to note that the underlying advantages conferred through this behavior extends beyond upregulating DNA synthesis 25. With that understanding, it is expected that there are many other regulatory pathways capable of regulating ASS1 expression. Recent studies have elucidated HIF1α as a potential regulator of ASS1 26. There is increasing evidence that HIF1α activation is capable of ASS1 repression through translational silencing. Primarily as a response mechanism to increasingly acidic/hypoxic environments, HIF1α is thought to utilize ASS1 inhibition to maintain pH homeostasis 26. Interestingly, the presence of hypoxia and low extracellular pH both appear to independently and synergistically enhance the silencing of ASS1. This was reported by Rogers et al who also hypothesized that the silencing of ASS1 occurs through multiple possible mechanisms such as the upregulated expression of miR-224-5p 27. By silencing ASS1, a subsequent increase in cellular pH buffering is reported as increasing intracellular concentration of urea, glutamine, and glutathione are also observed 27. Through the modulation of the urea cycle in this manner, Rogers et al additionally reports an inhibition of CAD. This understanding suggests that certain regulatory functions may hold similar effects on the urea cycle while operating in direct opposition to one another. A depiction of the metabolic shunting that occurs in ASS1 silenced GBMs can be seen in **Figure 2.**

Although the regulatory pathways responsible for ASS1 are not entirely understood, the correlation between the ASS1 negative phenotype of GBM and worse prognosis is not surprising 28. The metabolic reprogramming of the urea cycle has been shown to confer many advantages including enhanced anabolic function and cell survival 29. Importantly, these biochemical alterations warrant further investigation as there are many potential disadvantages to cancer cells demonstrating this phenotype. Most notably, the arginine auxotrophy displayed by ASS1 silencing cancer cells have demonstrated susceptibility to arginine deprivation therapies 30, 31. This has been well demonstrated in literature regarding leukemias such as acute myelogenous leukemia (AML) 32. By better understanding the underlying mechanisms responsible for this behavior, further opportunities to explore novel diagnostic and treatment modalities can be attained.

## Glutamate-Glutamine Cycle

A hallmark of many malignancies including GBMs is an extremely high rate of glutamine (Gln) consumption 33. Within non-proliferating cells, glutamine is a non-essential amino acid that is chiefly utilized for incorporation into proteins or as a nitrogen donor in the biosynthesis of amino acids and nucleotides 34. However, in highly proliferating cells glutamine consumption has been found to exceed the amount necessary for protein synthesis by as much as ten-fold, and cultured tumor cells require at least ten times more glutamine than any other amino acid 35, 36. The utilization is so extensive in some cancers that despite it being a non-essential amino acid, exogenous glutamine is required for tumor growth, a phenomenon termed “glutamine addiction” 33.

**Figure 3:**
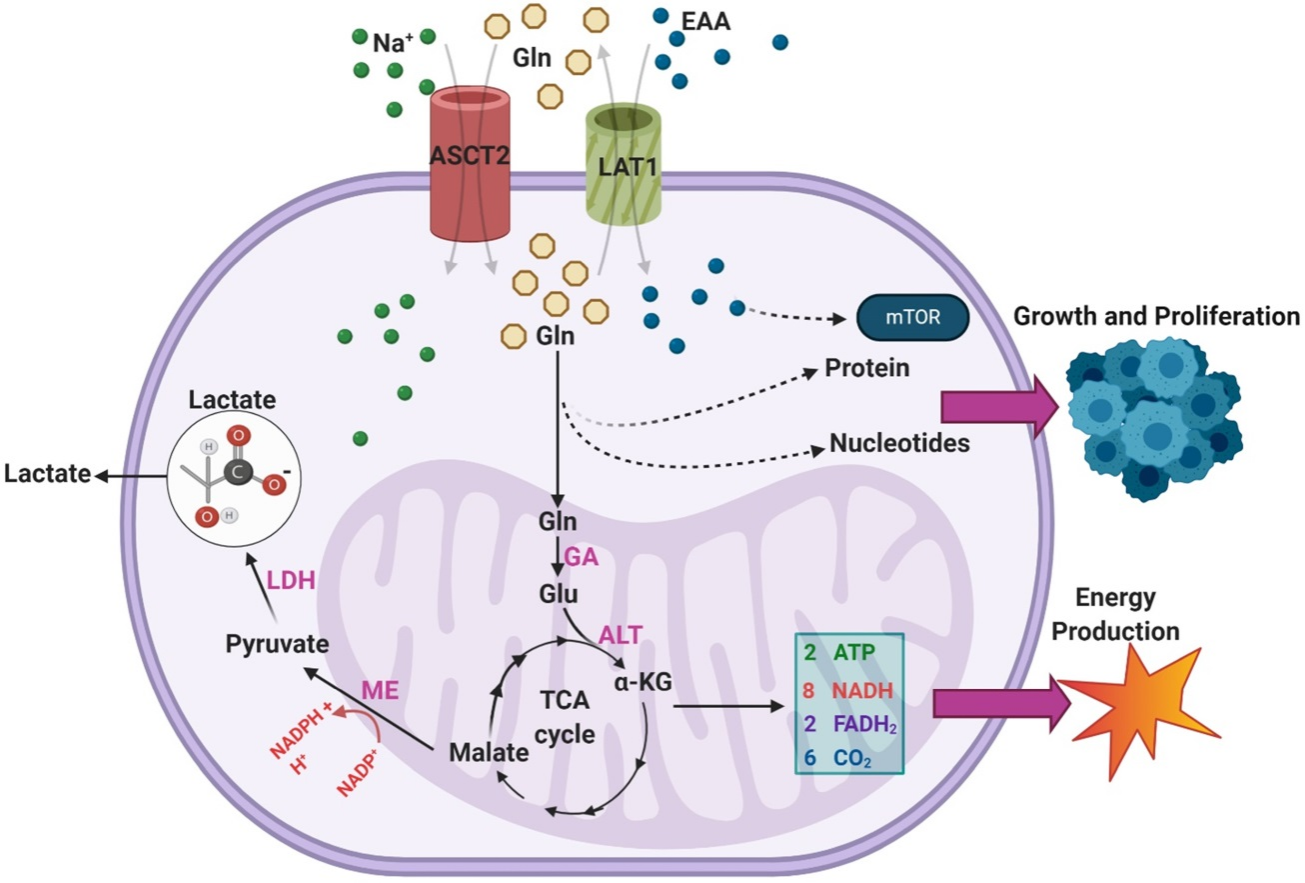
An overview of the pathways through which exogenous glutamine (Gln) is utilized within GBMs. Overexpression of ASCT2 in GBMs leads to enhanced Gln uptake so that it can be used as a substrate in the biosynthesis of nucleotides and proteins. The transport of Gln out of the cell in exchange for excitatory amino acids (EAA) via LAT1 is also upregulated in GBM, leading to enhanced mTOR signaling. Gln can also feed into the TCA cycle via a two-step process that results in the generation of α-KG. During the TCA cycle, a series of reactions converts α-KG into malate. Malate is then further processed to form pyruvate, a reaction which generates NADPH to support growth.

Malignant GBMs are among the cancer subtypes that exhibit increased glutamine uptake 37. Sidoryk et al observed significantly increased expression of mRNA transcripts of glutamine transporters system N transporter 3 (SNAT3) and alanine/serine/cysteine-preferring transporter 2 (ASCT2) in GBM samples 38. Overexpression of SNAT3 was confirmed at the protein level in GBM to verify the functional importance of this finding 38. Additionally, overexpression of ASCT2 was corroborated in an additional study by Dolinska et al. using a GBM derived C6 cell line 39. Within malignant cells, there are three main purposes for excess glutamine uptake and catabolism: generation of NADPH via anaplerosis in the TCA cycle, supporting a markedly increased production of glutamate, and facilitating uptake of essential amino acids (EAA) via the LAT1 antiporter 34. An overview of the various pathways through which exogenous glutamine is utilized in GBMs can be seen in **Figure 3.**

As seen, glutamine enters the TCA cycle as α-KG through a two-step process involving glutaminase (GA) and alanine aminotransferase (ALT) 34. From there it is eventually converted to malate and, through the activity of malic enzyme (ME), to pyruvate in a reaction that generates NADPH 34. This pyruvate is terminally converted to lactate by LDH, and the lactate is excreted 32. DeBerardinis et al. found that over 60% of glutamine was terminally converted to lactate in the SF188 glioblastoma cell line, indicating this pathway is highly active in GBM 36. The resultant NADPH was found to provide energy for fatty acid and nucleotide synthesis 36. Additionally, the majority of intracellular OAA in the cells was found to come from glutamine rather than glucose, suggesting that glutamine uptake is necessary to provide OAA for continued citrate synthase activity 36. This mechanism is particularly important in IDH-mutant GBMs, as glutamine metabolism replenishes TCA cycle intermediates that otherwise would not be present in high concentrations 36.

It has been demonstrated in multiple studies that the release of excitotoxic concentrations of glutamate via the cystine-glutamate antiporter promotes the growth of malignant GBMs 40-42. The resultant prolonged activation of N-methyl D-aspartate (NMDA) receptors in nearby neurons triggers an intracellular Ca^2+^ influx, inducing apoptosis 41. The destruction of peritumoral cells is thought to facilitate tumor invasion into surrounding tissues and provide malignant cells a competitive advantage for nutrient uptake 42.

Additionally, despite the high rate of release, GBM cells paradoxically show significantly (p < 0.001) reduced glutamate uptake compared to normal astrocytes 41. The C6 glioma cell line shows ubiquitous loss of the glutamate-aspartate transporter (GLAST), glutamate transporter 1 (GLT1) as well as frequent loss of excitatory amino acid carrier 1 (EAAC1) within subclonal populations 40. The reduced rate of glutamate uptake results in lower influx of Ca^2+^ within tumor cells, effectively shielding the cells from excitotoxicity 42. In fact, Ca^2+^ influx through AMPA receptors leads to the phosphorylation of AKT, promoting the growth and proliferation of malignant cells 43. The increased rate of glutamine uptake observed in GBM has been thought to contribute to endogenous glutamate production 38. This is corroborated by findings that increased GA activity directly correlates with tumor invasiveness 44.

With GBMs being so heavily dependent on glutamine uptake, alterations of glutamine metabolism or glutamate signaling make attractive therapeutic targets. Treatment of C6 glioma cells with the NMDA antagonist MK801 led to markedly decreased growth in glutamate-secreting tumors 40. The development of pharmacologic interventions targeting glutamine reservoirs in cancerous cells remains an ongoing and promising avenue of research 45, 46.

## One-Carbon Metabolism

One-carbon metabolism (OCM) is another system by which glioma maintain biosynthetic activity. In particular, OCM is a group of reactions occurring in the cytoplasm of mitochondria that are contained within the folate and methionine cycles and that provide methyl groups in order to allow for the generation of a host of metabolites including phospholipids, amino acids, and DNA 20, 47. In the folate cycle, folic acid is reduced to tetrahydrofolate (THF) via dyhydrofolate reductase, at which point THF can receive methyl groups from sources including units of serine and glycine in order to generate methyl-THF. Methyl-THF, as a one-carbon donator, is a highly useful cofactor involved in many processes, including the generation of S-adenosyl-methionine (SAM) via the methionine cycle 47-49. In the methionine cycle, methyl-THF donates a one-carbon unit to homocysteine to convert it to methionine, a reaction which is catalyzed by the enzyme methionine synthase. Methionine is then further acted upon by methionine adenosyltransferase, which allows for the production of SAM (which, through a series of reactions, can regenerate homocysteine, thus completing the cycle). SAM is a cofactor in many methylation reactions, and is thus vital to lipid, DNA, and protein production 49-51.

As important components of glioma metabolism, the folate and methionine cycles that is composed of one-carbon metabolism present promising targets for regulation of tumor metabolism. One investigation conducted by Xu and colleagues involved the use of the microRNA miR-940. It was found that miR-940 directly targets and inhibits methylenetetrahydrofolate dehydrogenase (MTHFD2), which is a key enzyme of folate one-carbon metabolism. Consequently, it was observed that overexpression of miR-940 not only decreased folate metabolism in cell samples, but also promoted apoptosis and inhibits invasion of glioma cells and, as such, poses a novel means of targeting tumor growth 52. Research into regulation of the methionine cycle as a means of curbing glioma metabolism is, unfortunately, less developed. However, some progress has, been made, as Palanichami and colleagues demonstrated that administration of exogenous methionine to GBM cells led to promotion and maintenance of said cells 53. As such, a means of inhibiting the amount of endogenous methionine available to glioma remains as a potentially fruitful avenue for further investigations.

## Autophagy

Autophagy is a highly conserved catabolic process by which a cell can digest and recycle its own cytosolic components 54. Paradoxically, this mechanism plays a vital role in cell death as well as cell survival. Non-selective autophagy of large cellular components such as mitochondria has been shown to contribute to cell apoptotic mechanisms 55. Conversely, in nutrient-poor conditions, autophagy can provide GBM tumor cells with essential metabolites that can be shunted towards a variety of cellular processes. Mammalian target of rapamycin (mTOR) dependent autophagy has been shown to recycle aging cell proteins and organelles that can be used by the TCA cycle for ATP generation 55. Sun et al. focused-on glioma autophagy and studied this process in the context of a transmembrane protein (CD133) that co-localized with both genes involved in autophagy (LC3, Beclin1, and ATG5) and lysosomes 56. This study demonstrated glioma cells that highly express CD133 have improved survival and decreased levels of apoptosis in starvation conditions when compared to CD133 negative glioma cells. Interestingly, this positive correlation was eliminated when both CD133-positive and CD133-negative cells were exposed to the anti-malarial chloroquine, which has been shown to disrupt autophagy 56, 57.

Along with enhancing cell survival, GBM tumors also use the resources provided through autophagy to enhance their metastatic capabilities. Within a subpopulation of glioma cancer stem cells (GSCs), it was established that the autophagy-associated genes DNA damage regulated autophagy modulator 1 (DRAM1) and p62 were closely associated with regulators of cell migration and invasion 58. GBM tumors come in many subtypes including the mesenchymal subtype, which shows the worst prognosis among patients. In *in-vitro* models, tumor cells that had the c-*MET* (a mesenchymal marker) gene silenced showed decreased invasive capabilities, which suggests that the mesenchymal subtype's prognosis is linked to the tumor's metastatic potential. Galavotti et al found high levels of DRAM1 and p62 expression in tumor cells of the mesenchymal subtype and demonstrated that mesenchymal tumors with high DRAM1 expression had further reductions in prognosis 58. This highlights a correlation between the autophagy capabilities of a tumor and its metastatic potential.

A discussion about autophagy would not be complete without discussing how the process contributes to tumor therapy resistance. GBM tumors are notorious for their high rates of drug resistance, and autophagy contributes to the tumor's ability to adapt to different therapies. The methylating agent temozolomide (TMZ) is a frequently used first-line therapy for GBM, and the development of TMZ resistance in GBM has been linked to autophagy 59, 60. TMZ-treated glioma cells were shown to increase expression of O^6^-methylguanine-DNA methyltransferase (MGMT), which is a primary trigger of autophagy 60. Knocking down MGMT expression *in-vitro* demonstrated increased susceptibility of glioma cells to TMZ treatment. Additionally, glioma cells that were treated with trehalose (TRE), an inducer of autophagy in an m-TOR independent pathway, exhibited increased resistance to TMZ 59. De-vascularization therapies such as bevacizumab also had limited efficacy on the treatment of GBM because they eventually promoted the process of hypoxia-induced autophagy 61. Under low oxygen conditions, HIF-1α acts to increase levels of a downstream target, Bcl-2 interacting protein 3 (BNIP3) in a process that promotes autophagy and increased cell survival in glioma (U87) and glioblastoma (T96G) cells. Hypoxia-induced autophagy was further demonstrated when bevacizumab-treated GBM cells were killed at a higher rate when they were co-treated with chloroquine *in vitro*. Additionally, *in vivo* experiments showed that bevacizumab-treated GBM cells with shRNA targeting and knocking down autophagy related gene 7 (ATG7), a gene essential for the formation of the autophagosome, exhibited 90% long-term survival 61. With these data in mind, combination therapies that decrease levels of autophagy have been shown to improve tumor response to chemotherapy. Notably, the combination of sirolimus/rapamycin (Rapa), an autophagy inducer, with chloroquine and TMZ caused increased glioma apoptosis *in-vitro* and inhibited growth *in-vivo* GBM xenografts 57. Blending Rapa and chloroquine alone showed increased cell apoptosis rates with Rapa maximizing cholesterol depletion in glioma cells, while chloroquine disrupts lysosomal membranes. The addition of TMZ augmented this process to further enhance glioma apoptosis and showed an increase in cell death when compared to the Rapa and chloroquine combination therapy. The efficacy of therapies such as TMZ, rapa, and chloroquine may be further augmented through combination therapies targeting the midkine (MDK) signaling axis 62. MDK is a neurotrophic factor that promotes the senescence of glioma initiating cells (GICs) through inhibiting autophagic degradation of the transcription factor SOX9 62. Inhibition of the MDK signaling axis was shown to decrease the self-renewal capacity of GBM cells, particularly when combined with TMZ 62. Promising targets in autophagy and other areas of tumor metabolism are continually being identified. A summary table of pertinent metabolic targets in GBM, including the ATG7 gene involved in autophagy, can be seen in **Table 1.**

## Future Directions / Conclusions

In summary, it can be seen that malignant GBMs utilize a variety of unconventional molecules to sustain growth. This endows the tumors with access to a far greater energy reservoir than healthy cells of the CNS, which almost exclusively rely on glucose for energy production 63. In addition to upregulating glycolysis, GBMs have been shown to use fatty acids, glutamine, metabolites such as folate and methionine (and their methylated derivatives), urea cycle metabolites, and the process of autophagy in excess to meet their high ATP demands. Since the uptake of these metabolites is disproportionately seen in malignant cells, targeting key regulatory molecules in their respective pathways represents a promising avenue in cancer therapy. Novel therapeutic targets aimed at altering tumor metabolism are continually being discovered and research in this field should be continued.

With a similar goal as the use of metabolically targeted therapies, the utility of dietary modifications in GBM treatment has become a topic of increasing interest. In particular, the ketogenic diet (KD) has been theorized to show benefit due to the resultant depletion of bodily glucose. Evidence on the efficacy of these diets in GBM is mixed 64-66. Using the orthotopic GL261 mouse glioma model, Ciusani et. al had found that mice fed with a KD showed greater survival compared to controls 64. This may be due to the fact that KDs attenuate GBM stemness and proliferation through enhancing ROS production 66. However, a recent study from Sperry et. al had shown that administration of a KD results in a compensatory increase in fatty acid oxidation 66. This compensation, combined with ketone body metabolism, was sufficient to sustain GBM growth and actually resulted in worse overall survival for KD mice in this study 66.

Research in both dietary modifications and metabolically targeted therapies are vital and should continue. Whether as standalone therapy or as sensitization agents administered alongside cytotoxic chemotherapy, targeted therapies against tumor metabolism show strong evidence to reduce GBM growth *in vitro*. By and large, the effects of these compounds *in vivo* remains to be seen, and so further studies will be necessary to fully delineate the adverse effects of metabolically targeted tumor therapies.

## Figures and Tables

**Table 1:** Promising therapeutic targets in tumor metabolism

Cellular Process	Target	Effects of Silencing
Glycolysis	PDK1	Reverses Warburg effect, promoting apoptosis and reducing tumor invasiveness 10.
Fatty Acid Oxidation	CPT-1	Etomoxir inhibits fatty acid oxidation and promotes intracellular ROS accumulation 19.
Urea Cycle	CAD	Reduces proliferation of ASS1 silenced cells
Glutamate-Glutamine	GRIN	NMDA receptor antagonist MK508 reduces tumor growth 40.
Autophagy	ATG7	Inhibits autophagosome formation 61.
